# Solution of the Fokker–Planck Equation by Cross Approximation Method in the Tensor Train Format

**DOI:** 10.3389/frai.2021.668215

**Published:** 2021-08-02

**Authors:** Andrei Chertkov, Ivan Oseledets

**Affiliations:** Skolkovo Institute of Science and Technology, Moscow, Russia

**Keywords:** fokker-planck equation, probability density function, tensor train format, cross approximation, chebyshev polynomial, ornstein-uhlenbeck process, dumbbell model

## Abstract

We propose the novel numerical scheme for solution of the multidimensional Fokker–Planck equation, which is based on the Chebyshev interpolation and the spectral differentiation techniques as well as low rank tensor approximations, namely, the tensor train decomposition and the multidimensional cross approximation method, which in combination makes it possible to drastically reduce the number of degrees of freedom required to maintain accuracy as dimensionality increases. We demonstrate the effectiveness of the proposed approach on a number of multidimensional problems, including Ornstein-Uhlenbeck process and the dumbbell model. The developed computationally efficient solver can be used in a wide range of practically significant problems, including density estimation in machine learning applications.

## 1 Introduction

Fokker–Planck equation (FPE) is an important in studying properties of the dynamical systems, and has attracted a lot of attention in different fields. In recent years, FPE has become widespread in the machine learning community in the context of the important problems of density estimation (Grathwohl et al., 2018) for neural ordinary differential equation (ODE) (Chen et al., 2018; Chen and Duvenaud, 2019), generative models (Kidger et al., 2021), etc.

Consider a stochastic dynamical system which is described by stochastic differential equation (SDE) of the form^1^

$$dx=f\left(x,t\right)\;dt+S\left(x,t\right)\;d\beta ,\;d\beta \;d\beta^{⊤}=Q\left(t\right)\;dt,\;x=x\left(t\right)\in {\mathbb{R}}^{d},$$ (1)

where dβ is a *q*-dimensional space-time white noise, **f** is a known *d*-dimensional vector-function and $S\in {\mathbb{R}}^{d\times q}$, $Q\in {\mathbb{R}}^{q\times q}$ are known matrices. The FPE for the corresponding probability density function (PDF) ρ(x,t) of the spatial variable x has the form

$$\frac{\partial \rho \left(x,t\right)}{\partial t}=\sum_{i=1}^{d}\sum_{j=1}^{d}\frac{\partial }{\partial x_{i}}\frac{\partial }{\partial x_{j}}\left[D_{ij}\left(x,t\right)\rho \left(x,t\right)\right]-\sum_{i=1}^{d}\frac{\partial }{\partial x_{i}}\left[f_{i}\left(x,t\right)\rho \left(x,t\right)\right],$$ (2)

where $D\left(x,t\right)=\frac{1}{2}S\left(x,t\right)Q\left(t\right)S^{⊤}\left(x,t\right)$ is a diffusion tensor.

One of the major complications in solution of the FPE is the high dimensionality of the practically significant computational problems. Complexity of using grid-based representation of the solution grows exponentially with *d*, thus some low-parametric representations are required. One of the promising directions is the usage of low-rank tensor methods, studied in (Dolgov et al., 2012). The equation is discretized on a tensor-product grid, such that the solution is represented as a *d*-dimensional tensor, and this tensor is approximated in the low-rank tensor train format (TT-format) (Oseledets, 2011). Even with such complexity reduction, the computations often take a long time. In this paper we propose another approach of using low-rank tensor methods for the solution of the FPE, based on its intimate connection to the dynamical systems.

The key idea can be illustrated for S=0, i.e. in the deterministic case. For this case the evolution of the PDF along the trajectory is given by the formula

$$\frac{\partial \rho \left(x,t\right)}{\partial t}=-\text{Tr}\left(\frac{\partial f\left(x,t\right)}{\partial x}\right)\;\rho \left(x,t\right),$$ (3)

where Tr( ⋅ ) is a trace operation for the matrix. Hence, to compute the value of ρ(x,t) at the specific point $x=\hat{x}$, it is sufficient to find a preimage ${\hat{x}}_{0}$ such that if it is used as an initial condition for eq. 1, then we arrive to $\hat{x}$. To find the preimage, we need to integrate the eq. 1 backwards in time, and then to find the PDF value, we integrate a system of eqs 1, 3. Since we can evaluate the value of ρ(x,t) at any $\hat{x}$, we can use the cross approximation method (CAM) (Oseledets and Tyrtyshnikov, 2010; Savostyanov and Oseledets, 2011; Dolgov and Savostyanov, 2020) in the TT-format to recover a supposedly low-rank tensor from its samples. In this way we do not need to have any compact representation of **f**, but only numerically solve the corresponding ODE. For S≠0 the situation is more complicated, but we develop a splitting and multidimensional interpolation schemes that allow us effectively recompute the values of the density from some time moment *t* to the next step t+h.

To summarize, main contributions of our paper are the following:

• we derive a formula to recompute the values of the PDF on each time step, using the second order operator splitting, Chebyshev interpolation and spectral differentiation techniques;
• we propose to use a TT-format and CAM to approximate the solution of the FPE which makes it possible to drastically reduce the number of degrees of freedom required to maintain accuracy as dimensionality increases;
• we implement FPE solver, based on the proposed approach, as a publicly available python code^2^, and we test our approach on several examples, including multidimensional Ornstein-Uhlenbeck process and dumbbell model, which demonstrate its efficiency and robustness.

## 2 Computation of the Probability Density Function

For ease of demonstration of the proposed approach, we suppose that the noise $\beta \in {\mathbb{R}}^{q}$ has the same dimension as the spatial variable $x\in {\mathbb{R}}^{d}$ (q=d), and the matrices in eq. 1 and eq. 2 have the form^3^

$$Q\left(t\right)\equiv I_{d},\;S\left(x,t\right)\equiv \sqrt{2\chi }I_{d},\;D\left(x,t\right)\equiv \chi I_{d},$$ (4)

where χ≥0 is a scalar diffusion coefficient. Then eqs 1, 2 can be rewritten in a more compact form

$$dx=f\left(x,t\right)\;dt+\sqrt{2\chi }d\beta ,\;d\beta \;d\beta^{⊤}=I_{d}\;dt,$$ (5)

$$\frac{\partial \rho }{\partial t}=\chi \text{Δ}\rho -\text{div}\left[f\left(x,t\right)\rho \right],$$ (6)

where *d*-dimensional spatial variable $x=x\left(t\right)\in \text{Ω}\subset {\mathbb{R}}^{d}$ has the corresponding PDF ρ(x,t) with initial conditions

$$x\left(0\right)=x_{0}∼\rho \left(x,0\right),\;\rho \left(x,0\right)=\rho_{0}\left(x\right).$$ (7)

To construct the PDF at some moment τ (τ>0) for the known initial distribution $\rho_{0}\left(x\right)$, we discretize eqs 5, 6 on the uniform time grid with *M* (M≥2) points

$$t_{m}=mh,\;h=\frac{\tau }{M-1},\;m=0,1,\ldots ,M-1,$$ (8)

and introduce the notation $x_{m}=x\left(t_{m}\right)$ for value of the spatial variable at the moment $t_{m}$ and $\rho_{m}\left(\;\cdot \;\right)=\rho \left(\;\cdot \;,t_{m}\right)$ for values of the PDF at the same moment.

### 2.1 Splitting Scheme

Let $\hat{V}$ and $\hat{W}$ be diffusion and convection operators from the eq. 6

$$\hat{V}v\equiv \chi \text{Δ}v,\;\hat{W}w\equiv -\text{div}\left[f\left(x,t\right)w\right],$$ (9)

then on each time step *m* (m=0,1,…,M−2) we can integrate equation

$$\frac{\partial \rho }{\partial t}=\left(\hat{V}+\hat{W}\right)\rho ,\;\rho \left(\cdot ,t_{m}\right)=\rho_{m}\left(\cdot \right),$$ (10)

on the interval $\left(t_{m},t_{m}+h\right)$, to find $\rho_{m+1}$ for the known value $\rho_{m}$ from the previous time step. Its solution can be represented in the form of the product of an initial solution with the matrix exponential

$$\rho_{m+1}=e^{h\left(\hat{V}+\hat{W}\right)}\rho_{m},$$ (11)

and if we apply the standard second order operator splitting technique (Glowinski et al., 2017), then

$$\rho_{m+1}\approx e^{\;\frac{h}{2}\hat{V}}e^{h\hat{W}}e^{\frac{h}{2}\hat{V}}\rho_{m},$$ (12)

which is equivalent to the sequential solution of the following equations

$$\frac{\partial v^{\left(1\right)}}{\partial t}=\chi \text{Δ}v^{\left(1\right)},\;v^{\left(1\right)}\left(\;\cdot \;,t_{m}\right)=\rho_{m}\left(\;\cdot \;\right),$$ (13)

$$\frac{\partial w}{\partial t}=-\text{div}\left[f\left(x,t\right)w\right],\;w\left(\;\cdot \;,t_{m}\right)=v^{\left(1\right)}\left(\;\cdot \;,t_{m}+\frac{h}{2}\right),$$ (14)

$$\frac{\partial v^{\left(2\right)}}{\partial t}=\chi \text{Δ}v^{\left(2\right)},\;v^{\left(2\right)}\left(\;\cdot \;,t_{m}\right)=w\left(\;\cdot \;,t_{m}+h\right),$$ (15)

with the final approximation of the solution $\rho_{m+1}\left(\;\cdot \;\right)=v^{\left(2\right)}\left(\;\cdot \;,t_{m}+\frac{h}{2}\right)$.

### 2.2 Interpolation of the Solution

To efficiently solve the convection eq. 14, we need the ability to calculate the solution of the diffusion eq. 13 at arbitrary spatial points, hence the natural choice for the discretization in the spatial domain are Chebyshev nodes, which makes it possible to interpolate the corresponding function on each time step by the Chebyshev polynomials (Trefethen, 2000).

We introduce the *d*-dimensional spatial grid $X^{\left(g\right)}$ as a tensor product of the one-dimensional grids^4^

$$x_{k}^{\left(g\right)}\in {\mathbb{R}}^{N_{k}},\;x_{k}^{\left(g\right)}\left[n_{k}\right]=\text{cos}\frac{\pi \cdot \left(n_{k}-1\right)}{N_{k}-1},\;n_{k}=1,2,\ldots ,N_{k},$$ (16)

where $N_{k}$ ($N_{k}\geq 2$) is a number of points along the *k*th spatial axis (k=1,2,…,d), and the total number of the grid points is $N=N_{1}\cdot N_{2}\cdot \ldots \cdot N_{d}$. Note that this grid can be also represented in the flatten form as a following matrix

$$X^{\left(g\right)}\in {\mathbb{R}}^{d\times N},\;X^{\left(g\right)}\left[k,n\right]=x_{k}^{\left(g\right)}\left[\mathrm{mind}\left(n\right)\left[k\right]\right],$$ (17)

where n=1,2,…,N, k=1,2,…,d and by $\mathrm{mind}\left(n\right)=[n_{1},n_{2},\ldots ,n_{d}^{⊤}$ we denoted an operation of construction of the multi-index from the flatten long index according to the big-endian convention

$$n=n_{d}+\left(n_{d-1}-1\right)N_{d}+\ldots +\left(n_{1}-1\right)N_{2}N_{3}\ldots N_{d}.$$ (18)

Suppose that we calculated PDF $\rho_{m}$ on some time step *m* (m≥0) at the nodes of the spatial grid $X^{\left(g\right)}$ [note that for the case m=0, the corresponding values come from the known initial condition $\rho_{0}\left(x\right)$]. These values can be collected as elements of a tensor^5^ ${ℛ}_{m}\in {\mathbb{R}}^{N_{1}\times N_{2}\times \ldots \times N_{d}}$ such that

$${ℛ}_{m}\left[n_{1},n_{2},\ldots ,n_{d}\right]=\rho_{m}\left(x_{1}^{\left(g\right)}\left[n_{1}\right],x_{2}^{\left(g\right)}\left[n_{2}\right],\ldots ,x_{d}^{\left(g\right)}\left[n_{d}\right]\right),$$ (19)

where $n_{k}=1,2,\ldots ,N_{k}$ (k=1,2,…,d).

Let us interpolate PDF $\rho_{m}$ via the system of orthogonal Chebyshev polynomials of the first kind

$$T_{0}\left(x\right)=1,\;T_{1}\left(x\right)=x,\;T_{k+1}\left(x\right)=2xT_{k}\left(x\right)-T_{k-1}\left(x\right)\;for\;k=1,2,\ldots ,$$ (20)

in the form of the naturally cropped sum

$$\begin{array}{l} \rho_{m}\left(x\right)\approx \tilde{\rho_{m}}\left(x\right)=\\ =\sum_{n_{1}=1}^{N_{1}}\sum_{n_{2}=1}^{N_{2}}\cdot \cdot \cdot \sum_{n_{d}=1}^{N_{d}}A_{m}\left[n_{1},n_{2},\ldots ,n_{d}\right]\;T_{n_{1}-1}\left(x_{1}\right)T_{n_{2}-1}\left(x_{2}\right)\ldots T_{n_{d}-1}\left(x_{d}\right), \end{array}$$ (21)

where $x=\left(x_{1},x_{2},\ldots ,x_{d}\right)$ is some spatial point and interpolation coefficients are elements of the tensor $A_{m}\in {\mathbb{R}}^{N_{1}\times N_{2}\times \ldots \times N_{d}}$. For construction of this tensor we should set equality in the interpolation nodes eq. 16

$$\begin{array}{l} \tilde{\rho_{m}}\left(x_{1}^{\left(g\right)}\left[n_{1}\right],\;\;x_{2}^{\left(g\right)}\left[n_{2}\right],\;\ldots ,\;x_{d}^{\left(g\right)}\left[n_{d}\right]\right)=\\ \rho_{m}\left(x_{1}^{\left(g\right)}\left[n_{1}\right],\;x_{2}^{\left(g\right)}\left[n_{2}\right],\;\ldots ,\;x_{d}^{\left(g\right)}\left[n_{d}\right]\right) \end{array}$$ (22)

for all combinations of $n_{k}=1,2,\ldots ,N_{k}$ (k=1,2,…,d).

Therefore the interpolation process can be represented as a transformation of the tensor ${ℛ}_{m}$ to the tensor $A_{m}$ according to the system of eq. 22. If the Chebyshev polynomials and nodes are used for interpolation, then a good way is to apply a fast Fourier transform (FFT) (Trefethen, 2000) for this transformation. However the exponential growth of computational complexity and memory consumption with the growth of the number of spatial dimensions makes it impossible to calculate and store related tensors for the multidimensional case in the dense data format. Hence in the next sections we present an efficient algorithm for construction of the tensor $A_{m}$ in the low-rank TT-format.

### 2.3 Solution of the Diffusion Equation

To solve the diffusion eqs 13, 15 on the Chebyshev grid, we discretize Laplace operator using the second order Chebyshev differential matrices [see, for example, (Trefethen, 2000)] $D_{k}\in {\mathbb{R}}^{N_{k}\times N_{k}}$ such that $D_{k}={\tilde{D}}_{k}{\tilde{D}}_{k}$, where for each spatial dimension k=1,2,…,d

$${\tilde{D}}_{k}\left[i,j\right]=\left\{\begin{array}{cc} \frac{2{\left(N_{k}-1\right)}^{2}+1}{6},\;i=j=1, & \\ \frac{-x_{k}^{\left(g\right)}\left[j\right]}{2\left(1-{\left(x_{k}^{\left(g\right)}\left[j\right]\right)}^{2}\right)},\;i=j=2,3,\ldots ,N_{k}-1, & \\ \frac{c_{i}}{c_{j}}\frac{{\left(-1\right)}^{i+j}}{x_{k}^{\left(g\right)}\left[i\right]-x_{k}^{\left(g\right)}\left[j\right]},\;i\neq j,\;i,j=2,3,\ldots ,N_{k}-1, & \\ -\frac{2{\left(N_{k}-1\right)}^{2}+1}{6},\;i=j=N_{k}, & \end{array}\right.$$ (23)

with $c_{i}=2$ if i=1 or $i=N_{k}$ and $c_{i}=1$ otherwise, and one dimensional grid points $x_{k}^{\left(g\right)}$ defined from eq. 16. Then discretized Laplace operator has the form^6^

$$\text{Δ}=D_{1}\;⊗\;I_{N_{2}}\;⊗\ldots ⊗\;I_{N_{d}}+I_{N_{1}}\;⊗\;D_{2}\;⊗\ldots \;⊗\;I_{N_{d}}+\ldots +I_{N_{1}}⊗I_{N_{2}}⊗\ldots ⊗D_{d}.$$ (24)

Let $V_{m}\in {\mathbb{R}}^{N_{1}\times N_{2}\times \ldots \times N_{d}}$ be the known initial condition for the diffusion equation on the time step *m* ($t_{m}=mh$), then for the solution $V_{m+\frac{1}{2}}$ at the moment $t_{m}+\frac{h}{2}$ we have

$$\mathrm{vec}\left(V_{m+\frac{1}{2}}\right)=e^{\frac{h}{2}\chi \text{Δ}}\mathrm{vec}\left(V_{m}\right),$$ (25)

where an operation vec(⋅) constructs a vector from the tensor by a standard reshaping procedure like eq. 18. And finally due to the well known property of the matrix exponential, we come to

$$\mathrm{vec}\left(V_{m\;+\;\frac{1}{2}}\right)=\left(e^{\frac{h}{2}\chi D_{1}}\;⊗\;e^{\frac{h}{2}\chi D_{2}}\;⊗\;\ldots \;⊗\;e^{\frac{h}{2}\chi D_{d}}\right)\mathrm{vec}\left(V_{m}\right).$$ (26)

If we can represent the initial condition $V_{m}$ in the form of Kronecker product of the one-dimensional tensors (for example, in terms of the TT-format in the form of the Kronecker products of the TT-cores, as will be presented below in this work), then we can efficiently evaluate the formula eq. 26 to obtain the desired approximation for solution $\mathrm{vec}\left(V_{m+\frac{1}{2}}\right)$.

### 2.4 Solution of the Convection Equation

Convection eq. 14 can be reformulated in terms of the FPE without diffusion part, when the corresponding ODE has the form

$$dx=f\left(x,t\right)\;dt,\;x=x\left(t\right)\in {\mathbb{R}}^{d},\;x∼\rho \left(x,t\right).$$ (27)

If we consider the differentiation along the trajectory of the particles, as was briefly described in the Introduction, then

$$\begin{array}{cc} {\left(\frac{\partial w}{\partial t}\right)}_{x=x\left(t\right)} & =\overset{d}{\underset{k=1}{\sum }}\frac{\partial w}{\partial x_{k}}\frac{\partial x_{k}}{\partial t}+\frac{\partial w}{\partial t}=\overset{d}{\underset{k=1}{\sum }}\frac{\partial w}{\partial x_{k}}\frac{\partial x_{k}}{\partial t}-\text{div}\left[fw\right]=\\ & =\overset{d}{\underset{k=1}{\sum }}\frac{\partial w}{\partial x_{k}}f_{k}-\overset{d}{\underset{k=1}{\sum }}\frac{\partial f_{k}}{\partial x_{k}}w-\overset{d}{\underset{k=1}{\sum }}f_{k}\frac{\partial w}{\partial x_{k}}=-\overset{d}{\underset{k=1}{\sum }}\frac{\partial f_{k}}{\partial x_{k}}w, \end{array}$$ (28)

where we replaced the term $\frac{\partial w}{\partial t}$ by the right hand side of eq. 14 and $\frac{\partial x_{k}}{\partial t}$ by the right hand side of the corresponding equation in eq. 27.

Hence equation for *w* may be rewritten in terms of the trajectory integration of the following system

$$\{\begin{array}{c} \frac{\partial x}{\partial t}=f\left(x,t\right),\\ \frac{\partial w}{\partial t}=-\text{Tr}\left(\frac{\partial f}{\partial x}\left(x,t\right)\right)w \end{array}$$ (29)

**FIGURE 1:**
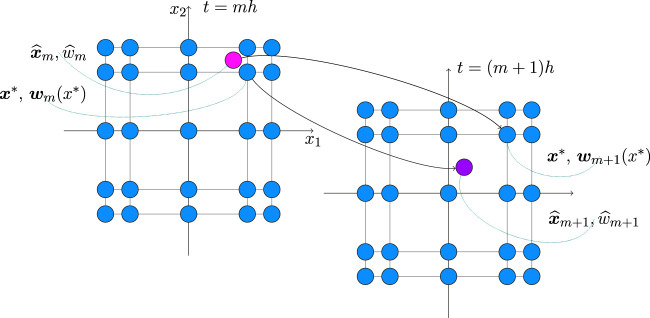
Evolution of the spatial variable and the corresponding PDF for two consecutive time steps related to the fixed Chebyshev grid in the case of two dimensions.

Let us integrate eq. 29 on a time step *m* (m=0,1,…,M−2). If we set any spatial grid point $x^{\text{*}}=X^{\left(g\right)}\left[:,n\right]$ (n=1,2,…,N) as initial condition for the spatial variable, then we’ll obtain solution ${\hat{w}}_{m+1}$ for some point ${\hat{x}}_{m+1}$ outside the grid (see Figure 1 with the illustration for the two-dimensional case). Hence we should firstly solve eq. 27 backward in time to find the corresponding spatial point ${\hat{x}}_{m}$ that will be transformed to the grid point $x^{\text{*}}$ by the step m+1. If we select this point ${\hat{x}}_{m}$ and the related value ${\hat{w}}_{m}=w\left({\hat{x}}_{m},t_{m}\right)$ as initial conditions for the system eq. 29, then its solution $w_{m+1}$ will be related to the point of interest $x^{\text{*}}$.

Note that, according to our splitting scheme, we solve the convection part eq. 14 after the corresponding diffusion eq. 13, and hence the initial condition $w_{m}$ is already known and defined as a tensor $W_{m}\in {\mathbb{R}}^{N_{1}\times N_{2}\times \ldots \times N_{d}}$ on the Chebyshev spatial grid. Using this tensor, we can perform interpolation according to the formula eq. 22 and calculate the tensor of interpolation coefficients $A_{m}$. Then we can evaluate the approximated value at the point ${\hat{x}}_{m}$ as $\tilde{w_{m}}\left({\hat{x}}_{m}\right)$ according to eq. 21.

Hence our solution strategy for convection equation is the following. For the given spatial grid point $x^{\text{*}}=X^{\left(g\right)}\left[:,n\right]$ we integrate equation

$$\frac{\partial x}{\partial t}=f\left(x,t\right),\;x\left(t_{m+1}\right)=x\text{*},$$ (30)

backward in time to find the corresponding point ${\hat{x}}_{m}=x\left(t_{m}\right)$. Then we find the value of *w* at this point, using interpolation $\tilde{w_{m}}$, and then we solve the system eq. 29 on the time interval $\left(t_{m},t_{m}+h\right)$ with initial condition $\left({\hat{x}}_{m},\tilde{w_{m}}\left({\hat{x}}_{m}\right)\right)$ to obtain the value $w_{m+1}$ at the point $x^{\text{*}}$. The described process should be repeated for each grid point (n=1,2,…,N) and, ultimately, we’ll obtain a tensor $W_{m+1}\in {\mathbb{R}}^{N_{1}\times N_{2}\times \ldots \times N_{d}}$ which is the approximated solution of convection part eq. 14 of the splitting scheme on the Chebyshev spatial grid.

An important contribution of this paper is an indication of the possibility and a practical implementation of the usage of the multidimensional CAM in the TT-format to recover a supposedly low-rank tensor $W_{m+1}$ from computations on only a part of specially selected spatial grid points. This scheme will be described in more details later in the work after setting out the fundamentals of the TT-format.

## 3 Low-Rank Representation

There has been much interest lately in the development of data-sparse tensor formats for high-dimensional problems. A very promising tensor format is provided by the tensor train (TT) approach (Oseledets and Tyrtyshnikov, 2009; Oseledets, 2011), which was proposed for compact representation and approximation of high-dimensional tensors. It can be computed via standard decompositions (such as SVD and QR-decomposition) but does not suffer from the curse of dimensionality^7^.

In many analytical considerations and practical cases a tensor is given implicitly by a procedure enabling us to compute any of its elements, so the tensor appears rather as a black box. For example, to construct the convection part of PDF (i.e., the tensor $W_{m}$ introduced above), we should compute the corresponding function for all possible sets of indices. This process requires an extremely large number of operations and can be time-consuming, so it may be useful to find some suitable low-parametric approximation of this tensor using only a small portion of all tensor elements. CAM (Oseledets and Tyrtyshnikov, 2010) which is a widely used method for approximation of high-dimensional tensors looks appropriate for this case.

In this section we describe the properties of the TT-format and multidimensional CAM that are necessary for efficient solution of our problem, as well as the specific features of the practical implementation of interpolation by the Chebyshev polynomials in terms of the TT-format and CAM.

### 3.1 Tensor Train Format

A tensor $ℛ\in {\mathbb{R}}^{N_{1}\times N_{2}\times \ldots \times N_{d}}$ is said to be in the TT-format (Oseledets, 2011), if its elements are represented by the formula

$$\begin{array}{l} ℛ\left[n_{1},n_{2},\ldots ,n_{d}\right]=\sum_{r_{1}=1}^{R_{1}}\sum_{r_{2}=1}^{R_{2}}\cdot \cdot \cdot \sum_{r_{d-1}=1}^{R_{d-1}}G_{1}\left[1,n_{1},r_{1}\right]G_{2}\left[r_{1},n_{2},r_{2}\right]\ldots \\ \;\;\;\;\;\;\;\;\;\;\;\;\;\;\;\;\;\;\;\;\;\;\;\;\;\;\;\;\;\;\;\;\;\;\;\;\;\;\;\;\;\;\;\;\;\;\;\;\;\;\;\;\;\;\;G_{d-1}\left[r_{d-2},n_{d-1},r_{d-1}\right]G_{d}\left[r_{d-1},n_{d},1\right] \end{array}$$ (31)

where $n_{k}=1,2,\ldots ,N_{k}$ (k=1,2,…,d), three-dimensional tensors $G_{k}\in {\mathbb{R}}^{R_{k-1}\times N_{k}\times R_{k}}$ are named TT-cores, and integers $R_{0},R_{1},\ldots ,R_{d}$ (with convention $R_{0}=R_{d}=1$) are named TT-ranks. The latter formula can be also rewritten in a more compact form

$$ℛ\left[n_{1},n_{2},\ldots ,n_{d}\right]=G_{1}\left(n_{1}\right)G_{2}\left(n_{2}\right)\ldots G_{d}\left(n_{d}\right),$$ (32)

where $G_{k}\left(n_{k}\right)=G_{k}\left[:,n_{k},:\right]$ is an $R_{k-1}\times R_{k}$ matrix for each fixed $n_{k}$ (since $R_{0}=R_{d}=1$, the result of matrix multiplications in eq. 32 is a scalar). And a vector form of the TT-decomposition looks like

$$\mathrm{vec}\left(ℛ\right)=\sum_{r_{1}=1}^{R_{1}}\sum_{r_{2}=1}^{R_{2}}\cdot \cdot \cdot \sum_{r_{d-1}=1}^{R_{d-1}}G_{1}\left[1,:,r_{1}\right]⊗G_{2}\left[r_{1},:,r_{2}\right]⊗\ldots ⊗G_{d}\left[r_{d-1},:,1\right],$$ (33)

where the slices of the TT-cores $G_{k}$ are vectors of length $N_{k}$ (k=1,2,…,d).

The benefit of the TT-decomposition is the following. Storage of the TT-cores $G_{1},G_{2},\ldots ,G_{d}$ requires less or equal than $d\times {\text{max}}_{1\leq k\leq d}\left(N_{k}R_{k}^{2}\right)$ memory cells (instead of $N=N_{1}N_{2}\ldots N_{d}∼N_{0}^{d}$ cells for the uncompressed tensor, where $N_{0}$ is an average size of the tensor modes), and hence the TT-decomposition is free from the curse of dimensionality if the TT-ranks are bounded.

The detailed description of the TT-format and linear algebra operations in terms of this format^8^ is given in works (Oseledets and Tyrtyshnikov, 2009; Oseledets, 2011). It is important to note that for a given tensor $\hat{ℛ}$ in the full format, the TT-decomposition (compression) can be performed by a stable TT-SVD algorithm. This algorithm constructs an approximation ℛ in the TT-format to the given tensor $\hat{ℛ}$ with a prescribed accuracy $\epsilon_{TT}$ in the Frobenius norm^9^

$${\left\|ℛ-\hat{ℛ}\right\|}_{F}\leq \epsilon_{TT}\cdot {\left\|\hat{ℛ}\right\|}_{F},$$ (34)

but a procedure of the tensor approximation in the full format is too costly, and is even impossible for large dimensions due to the curse of dimensionality. Therefore more efficient algorithms like CAM are needed to quickly construct the tensor in the low rank TT-format.

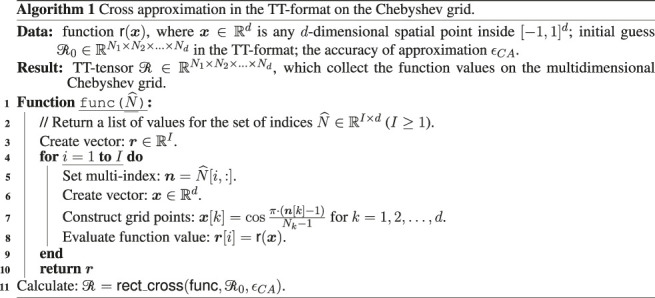

### 3.2 Cross Approximation Method

The CAM allows to construct a TT-approximation of the tensor with prescribed accuracy $\epsilon_{CA}$, using only part of the full tensor elements. This method is a multi-dimensional analogue of the simple cross approximation method for the matrices (Tyrtyshnikov, 2000) that allows one to approximate large matrices in $O\left(N_{0}R^{2}\right)$ time by computing only $O\left(N_{0}R\right)$ elements, where $N_{0}$ is an average size of the matrix modes and *R* is the rank of the matrix. The CAM and the TT-format can significantly speed up the computation and reduce the amount of consumed memory as will be illustrated in the next sections on the solution of the model equations.

The CAM constructs a TT-approximation ℛ to the tensor $\hat{ℛ}$, given as a function $f\left(n_{1},n_{2},\ldots ,n_{d}\right)$, that returns the $\left(n_{1},n_{2},\ldots ,n_{d}\right)$ th entry of $\hat{ℛ}$ for a given set of indices. This method requires only

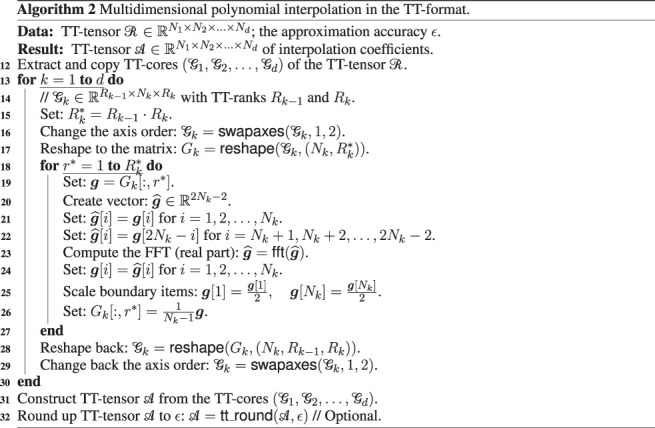

$O\left(d\times {\text{max}}_{1\leq k\leq d}\left(N_{k}R_{k}^{3}\right)\right)$ operations for the construction of the approximation with a prescribed accuracy $\epsilon_{CA}$, where $R_{0},R_{1},\ldots ,R_{d}$ ($R_{0}=R_{d}=1$) are TT-ranks of the tensor ℛ [see detailed discussion of the CAM in (Oseledets and Tyrtyshnikov, 2010)]. It should be noted that TT-ranks can depend on the value of selected accuracy $\epsilon_{CA}$, but for a wide class of practically interesting tasks the TT-ranks are bounded or depend polylogarithmically on $\epsilon_{CA}$ [(Oseledets, 2010; Oseledets, 2011) for more details and examples].

In Algorithm 1 the description of the process of construction of the tensor in the TT-format on the Chebyshev grid by the CAM is presented (we’ll call it as a function $\mathrm{cros}s_{X}\left(\;\cdot \;\right)$ below). We prepare function func, which transforms given indices into the spatial grid points and return an array of the corresponding values of the target r( ⋅ ). Then this function is passed as an argument to the standard rank adaptive method tt_rectcross from the ttpy package. The CAM is described in more detail in the original papers (Oseledets and Tyrtyshnikov, 2010; Savostyanov and Oseledets, 2011), as well as in a recent work (Dolgov and Savostyanov, 2020), which formulates a computationally efficient parallel implementation of the algorithm.

### 3.3 Multidimensional Interpolation

As was discussed in the previous sections, we discretize the FPE on the multidimensional Chebyshev grid and interpolate solution of the first diffusion equation in the splitting scheme eq. 13 by the Chebyshev polynomials to obtain its values on custom spatial points (different from the grid nodes) and then perform efficient trajectory integration of the convection eq. 14.

The desired interpolation may be constructed from solution of the system of eq. 22 in terms of the FFT (Trefethen, 2000), but for the high dimension numbers we have the exponential growth of computational complexity and memory consumption, hence it is very promising to construct tensor of the nodal values and the corresponding interpolation coefficients in the TT-format.

Consider a TT-tensor $ℛ\in {\mathbb{R}}^{N_{1}\times N_{2}\times \ldots \times N_{d}}$ with the list of TT-cores $\left[G_{1},G_{2},\ldots G_{d}\right]$, which collects PDF values on the nodes of the Chebyshev grid at some time step (the related function is r(x), and this tensor is obtained, for example, by the CAM or according to TT-SVD procedure from the tensor in the full format). Then the corresponding TT-tensor $A\in {\mathbb{R}}^{N_{1}\times N_{2}\times \ldots \times N_{d}}$ of interpolation coefficients with the TT-cores $\left[{\tilde{G}}_{1},{\tilde{G}}_{2},\ldots {\tilde{G}}_{d}\right]$ can be constructed according to the scheme, which is presented in Algorithm 2 [we’ll call it as a function interpolate( ⋅ ) below].

In this Algorithm we use standard linear algebra operations swapaxes and reshape, which rearrange the axes and change the dimension of the given tensor respectively, function fft for construction of the one-dimensional FFT for the given vector, and function tt_round from the ttpy package, which round the given tensor to the prescribed accuracy ϵ. Note that the inner loop in Algorithm 2 for $r^{\text{*}}$ may be replaced by the vectorized computations of the corresponding two-dimensional FFT.

For the known tensor A we can perform a fast computation of the function value at any given spatial point $x=[x_{1},x_{2},\ldots ,x_{d}^{⊤}]$ by a matrix product of the convolutions of the TT-cores of A with appropriate column vectors of Chebyshev polynomials

$$\begin{array}{c} r\left(x\right)\approx \overset{R_{1}}{\underset{r_{1}=1}{\sum }}\overset{R_{2}}{\underset{r_{2}=1}{\sum }}\ldots \overset{R_{d-1}}{\underset{r_{d-1}=1}{\sum }}\left(\overset{N_{1}}{\underset{n_{1}=1}{\sum }}{\tilde{G}}_{1}\left[1,n_{1},r_{1}\right]T_{n_{1}-1}\left(x_{1}\right)\right)\\ \left(\overset{N_{2}}{\underset{n_{2}=1}{\sum }}{\tilde{G}}_{2}\left[r_{1},n_{2},r_{2}\right]T_{n_{2}-1}\left(x_{2}\right)\right)\ldots \left(\overset{N_{d}}{\underset{n_{d}=1}{\sum }}{\tilde{G}}_{d}\left[r_{d-1},n_{d},1\right]T_{n_{d}-1}\left(x_{d}\right)\right), \end{array}$$ (35)

We’ll call the corresponding function as inter_eval(A,X) below. This function constructs a list of r( ⋅ ) values for the given set of *I* points $X\in {\mathbb{R}}^{d\times I}$ (I≥1), using interpolation coefficients A and sequentially applying the formula eq. 35 for each spatial point.

## 4 Detailed Algorithms

In Algorithms 3, 4 and 5 we combine the theoretical details discussed in the previous sections of this work and present the final calculation scheme for solution of the multidimensional FPE in the TT-format, using CAM (function $\mathrm{cros}s_{X}$, Algorithm 1)^10^ and interpolation by the Chebyshev polynomials (function interpolate from Algorithm 2 that constructs interpolation coefficients and function inter_eval that evaluates interpolation result at given points according to the formula eq. 35).

We denote by einsum the standard linear algebra operation that evaluates the Einstein summation convention on the operands (see, for example, the numpy python package). Function vstack stack arrays in sequence vertically, function $\mathrm{ode}\_\mathrm{solve}\left(\mathrm{rhs},t_{1},t_{2},Y_{0}\right)$ (where $t_{1}$ and $t_{2}$ are initial and final times, rhs is the right hand side of equations, and matrix $Y_{0}$ collects initial conditions) solves a system of ODE with vectorized initial condition by the one step of the fourth order Runge-Kutta method.

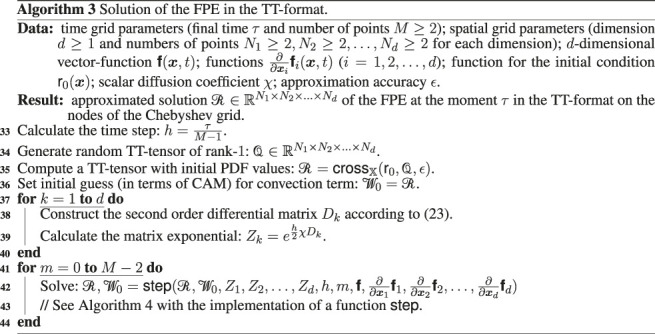

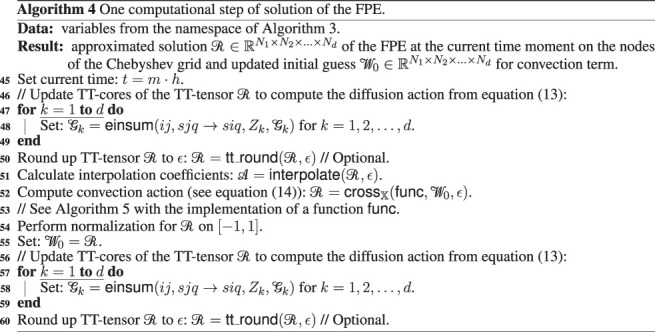

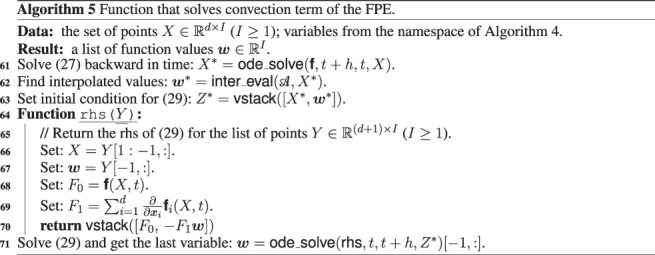

## 5 Numerical Examples

In this section we illustrate the proposed computational scheme, which was presented above, with the numerical experiments. All calculations were carried out in the Google Colab cloud interface^11^ with the standard configuration (without GPU support).

Firstly we consider an equation with a linear convection term—Ornstein-Uhlenbeck process (OUP) (Vatiwutipong and Phewchean, 2019) in one, three and five dimensions. For the one-dimensional case, which is presented for convention, we only solve equation using the dense format (not TT-format), hence the corresponding results are used to verify the general correctness and convergence properties of the proposed algorithm, but not its efficiency. In the case of the multivariate problems we use the proposed tensor based solver, which operates in accordance with the algorithm described above. To check the results of our computations, we use the known analytic stationary solution for the OUP, and for the one-dimensional case we also perform comparison with constructed analytic solution at any time moment.

Then we consider more complicated dumbbell problem (Venkiteswaran and Junk, 2005) which may be represented as a three-dimensional FPE with a nonlinear convection term. For this case we consider the Kramer expression and compare our computation results with the results from another works for the same problem.

In the numerical experiments we consider the spatial region Ω such that PDF is almost vanish on the boundaries $\rho \left(x,t\right){|}_{\partial \text{Ω}}\approx 0$, and the initial condition is selected in the form of the Gaussian function

$$\rho \left(x,0\right)=\rho_{0}\left(x\right)={\left(2\pi s\right)}^{-\frac{d}{2}}\text{exp}\left[-\frac{1}{2s}||x|{|}^{2}\right],\;s\in \mathbb{R},\;s>0,$$ (36)

where parameter *s* is selected as s=1. To estimate the accuracy of the obtained PDF (ρ) we use the relative 2-norm of deviation from the exact value ($\rho_{exact}$)

$$e=\frac{||\rho -\rho_{exact}|{|}_{2}}{|\rho_{exact}|{|}_{2}}.$$ (37)

We compute the value $\rho_{exact}$ through the given function, using a CAM with an accuracy parameter two orders of magnitude higher, than the one that was used in the solver.

### 5.1 Numerical Solution of the Ornstein-Uhlenbeck Process

Consider FPE of the form eq. 6 in the *d*-dimensional case with

$$f\left(x,t\right)=A\left(\mu -x\left(t\right)\right),\;\chi =\frac{1}{2},\;x\in \text{Ω}={\left[x_{min},x_{max}\right]}^{d},\;t\in \left[0,\tau \right],$$ (38)

where $A\in {\mathbb{R}}^{d\times d}$ is invertible real matrix, $\mu \in {\mathbb{R}}^{d}$ is the long-term mean, $x_{min}\in \mathbb{R}$ and $x_{max}\in \mathbb{R}$ ($x_{min}<x_{max}$), τ∈ℝ (τ>0). This equation is a well known multivariate OUP with the following properties [see for example (Singh et al., 2018; Vatiwutipong and Phewchean, 2019)]:

• mean vector is

$$M\left(t,x_{0}\right)=e^{-At}x_{0}+\left(I_{d}-e^{-At}\right)\mu ;$$ (39)

• covariance matrix is

$$\text{Σ}\left(t\right)=\int_{0}^{t}e^{A\left(s-t\right)}SS^{⊤}e^{A^{⊤}\left(s-t\right)}d\;s,$$ (40)

and, in our case as noted above $S=\sqrt{2\chi }I_{d}$;

• transitional PDF is

$$\rho \left(x,t,x_{0}\right)=\frac{\text{exp}\left[-\frac{1}{2}{\left(x-M\left(t,x_{0}\right)\right)}^{⊤}{\text{Σ}}^{-1}\left(t\right)\left(x-M\left(t,x_{0}\right)\right)\right]}{\sqrt{\left|2\pi \text{Σ}\left(t\right)\right|}};$$ (41)

• stationary solution is

$$\rho_{st}\left(x\right)=\frac{\text{exp}\left[-\frac{1}{2}x^{⊤}W^{-1}x\right]}{\sqrt{{\left(2\pi \right)}^{d}det\left(W\right)}},$$ (42)

where matrix $W\in {\mathbb{R}}^{d\times d}$ can be found from the following equation

$$AW+WA^{⊤}=2\chi I_{d};$$ (43)

• the (multivariate) OUP at any time is a (multivariate) normal random variable;
• the OUP is mean-reverting (the solution tends to its long-term mean μ as time *t* tends to infinity) if all eigenvalues of *A* are positive (if A>0 in the one-dimensional case).

#### 5.1.1 One-Dimensional Process

Let consider the one-dimensional (d=1) OUP with

$$A=1,\;\mu =0,\;x_{min}=-5,\;x_{max}=5,\;\tau =10.$$ (44)

We can calculate the analytic solution in terms of only spatial variable and time via integration of the transitional PDF eq. 41

$$\rho \left(x,t\right)=\int_{-\infty }^{\infty }\rho \left(x,t,x_{0}\right)\rho_{0}\left(x_{0}\right)\;dx_{0}.$$ (45)

Accurate computations lead to the following formula

$$\rho \left(x,t\right)=\frac{1}{\sqrt{2\pi \left(\text{Σ}\left(t\right)+se^{-2At}\right)}}\text{exp}\left[-\frac{x^{2}}{2\left(\text{Σ}\left(t\right)+se^{-2At}\right)}\right],$$ (46)

where Σ(t) is defined by eq. 40 and for the one-dimensional case may be represented in the form

$$\text{Σ}\left(t\right)=\frac{1-e^{-2At}}{2A}.$$ (47)

Using the formulas eq. 42 and eq. 43 we can represent a stationary solution for the one-dimensional case in the explicit form

$$\rho_{stat}\left(x\right)=\sqrt{\frac{A}{\pi }}e^{-Ax^{2}}.$$ (48)

**FIGURE 2:**
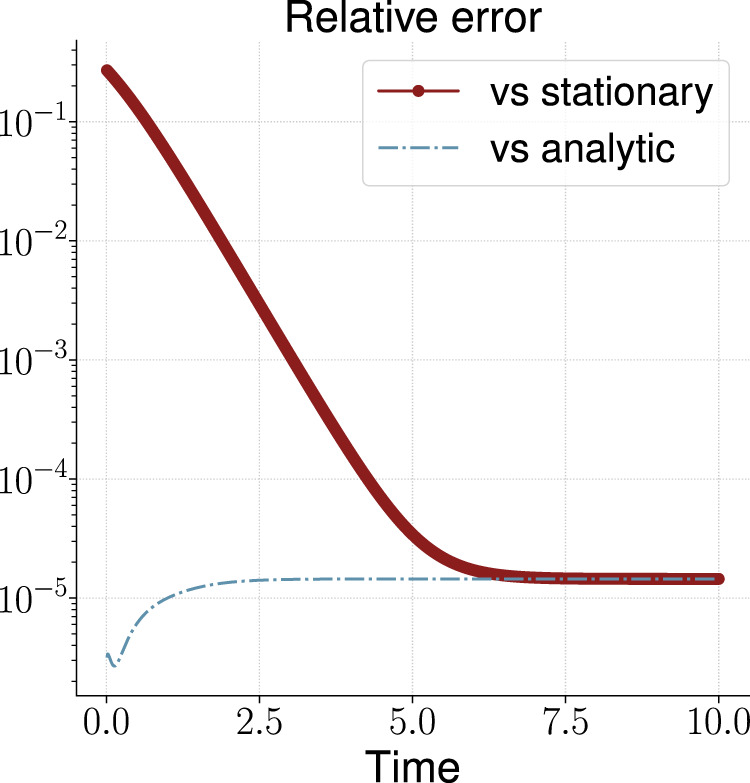
Relative error of the calculated solution vs known analytic and stationary solutions for the one-dimensional OUP.

We perform computation for $N_{1}=50$ spatial points and M=1000 time points and compare the numerical solution with the known analytic eq. 46 and stationary eq. 48 solution. In the Figure 2 we present the corresponding result. Over time, the error of the numerical solution relative to the analytical solution first increases slightly, and then stabilizes at approximately $10^{-5}$. At the same time, the numerical solution approaches the stationary one, and the corresponding error at large times also becomes approximately $10^{-5}$. Note that the time to build the solution was about 5 s.

#### 5.1.2 Three-Dimensional Process

Our next example is the three-dimensional (d=3) OUP with the following parameters

$$A=\left[\begin{array}{ccc} 1.5 & 1 & 0\\ 0 & 1 & 0\\ 0.5 & 0.3 & 1 \end{array}\right],\;\mu =0,\;x_{min}=-5,\;x_{max}=5,\;\tau =5.$$ (49)

When carrying out numerical calculation, we select $10^{-4}$ as the accuracy of the CAM, 100 as a total number of time points and 30 as a number of points along each of the spatial dimensions. The computation result is compared with the stationary solution eq. 42 which was obtained as solution of the related matrix eq. 43 by a standard solver for Lyapunov equation.

**FIGURE 3:**
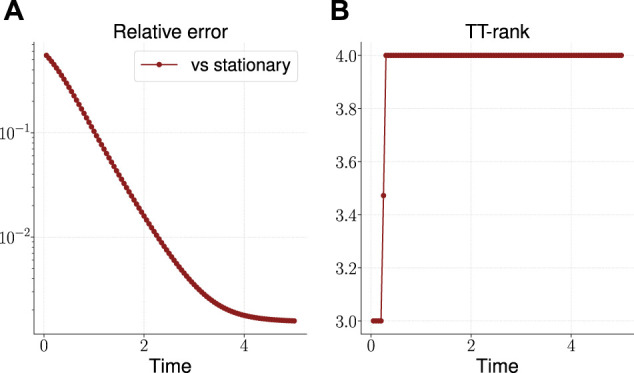
Relative error of the calculated solution vs known stationary solution **(A)** and the effective TT-rank **(B)** for the three-dimensional OUP.

The result is shown in Figure 3. As can be seen, the TT-rank^12^ remains limited, and the accuracy of the solution over time grows, reaching $10^{-3}$ by the time t=5. The time to build the solution was about 26 s.

To evaluate the efficiency of the proposed algorithm in the TT-format, we also solve these three-dimensional OUP, using dense format (as for the one-dimensional case, all arrays are presented in its full form). The corresponding calculation took about 376 s, so in this case we have an acceleration of calculations by more than an order of magnitude.

#### 5.1.3 Five-Dimensional Process

This multidimensional case is considered in the same manner as the previous one. We select the following parameters

$$A=\left[\begin{array}{ccccc} 1.5 & 0 & 0 & 0 & 0\\ 0 & 1 & 0 & 0 & 0\\ 0 & 0 & 1 & 0 & 0\\ 0 & 0 & 0 & 1 & 0\\ 0.5 & 0.3 & 0.2 & 0 & 1 \end{array}\right],\;\mu =0,\;x_{min}=-5,\;x_{max}=5,\;\tau =5.$$ (50)

We select the same values as in the previous example for the CAM accuracy ($10^{-4}$), the number of time points (100) and the number of spatial points (30), and compare result of the computation with the stationary solution from eq. 42 and eq. 43.

**FIGURE 4:**
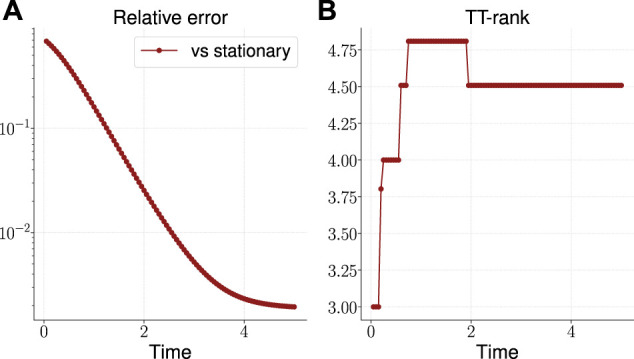
Relative error of the calculated solution vs known stationary solution **(A)** and the effective TT-rank **(B)** for the five-dimensional OUP.

The results are presented on the plots on Figure 4. The TT-rank of the solution remains limited and reaches the value 4.5 at the end time step, and the solution accuracy reaches almost $10^{-3}$. The time to build the solution was about 100 s.

### 5.2 Numerical Solution of the Dumbbell Problem

Now consider a more complex non-linear example corresponding to the three-dimensional (d=3) dumbbell model of the form eq. 6 with^13^

$$f\left(x,t\right)=Ax-\frac{1}{2}\nabla \phi ,\;A=\beta \left[\begin{array}{ccc} 0 & 1 & 0\\ 0 & 0 & 0\\ 0 & 0 & 0 \end{array}\right],\;\phi =\frac{||x|{|}^{2}}{2}+\frac{\alpha }{p^{3}}e^{-\frac{||x|{|}^{2}}{2p^{2}}},$$ (51)

where

$$\chi =\frac{1}{2},\;x\in \text{Ω}={\left[-10,10\right]}^{3},\;t\in \left[0,10\right],\;\alpha =0.1,\;\beta =1,\;p=0.5.$$ (52)

Making simple calculations (taking into account the specific form of the matrix *A*), we get explicit expressions for the function and the required partial derivatives (k=1,2,3)

$$f=\beta \left[\begin{array}{c} x_{2}\\ 0\\ 0 \end{array}\right]-\frac{1}{2}x+\frac{\alpha }{2p^{5}}e^{-\frac{||x|{|}^{2}}{2p^{2}}}x,$$ (53)

$$\frac{\partial f_{k}}{\partial x_{k}}=-\frac{1}{2}+\frac{\alpha }{2p^{5}}e^{-\frac{||x|{|}^{2}}{2p^{2}}}-\frac{\alpha }{2p^{7}}e^{-\frac{||x|{|}^{2}}{2p^{2}}}x_{k}^{2}.$$ (54)

Next, we consider the Kramer expression

τ(t)=∫ρ(x,t)[x⊗∇ϕ]dx, (55)

and as the values of interest (as in the works (Venkiteswaran and Junk, 2005; Dolgov et al., 2012)) we select

$$\psi \left(t\right)=\frac{\tau_{11}\left(t\right)-\tau_{22}\left(t\right)}{\beta^{2}}=\frac{1}{\beta^{2}}\rho \left(x,t\right)\left(x_{1}\frac{\partial \phi }{\partial x_{1}}-x_{2}\frac{\partial \phi }{\partial x_{2}}\right),$$ (56)

$$\eta \left(t\right)=\frac{\tau_{12}\left(t\right)}{\beta }=\frac{1}{\beta }\rho \left(x,t\right)x_{1}\frac{\partial \phi }{\partial x_{2}}.$$ (57)

During the calculations we used the following solver parameters:

• the accuracy of the CAM is $10^{-5}$;
• the number of time grid points is 100;
• the number of grid points along each of the spatial dimensions is 60.

**FIGURE 5:**
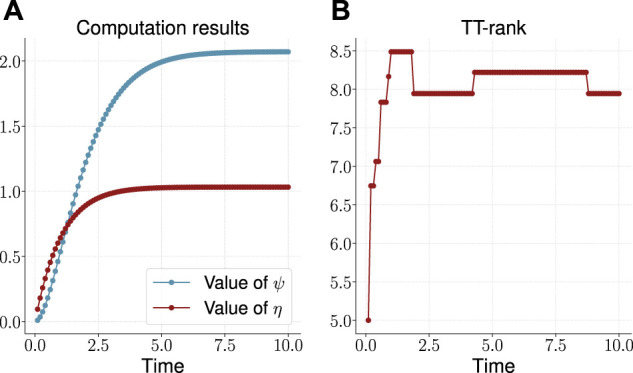
Computed values **(A)** and the effective TT-rank **(B)** for the three-dimensional dumbbell problem.

The results are presented on the plots on Figure 5. The time to build the solution was about 200 s (also additional time was required to calculate the values ψ(t) and η(t) from eq. 56 and eq. 57 respectively). As can be seen, the TT-rank remains limited, and its stationary value is about 8. We compared the obtained stationary values of the ψ(t) and η(t) variables:

ψ(t=10)=2.0707, η(t=10)=1.0318, (58)

with the corresponding results from (Dolgov et al., 2012)^14^, and we get the following values for relative errors

$$\epsilon_{\psi }=1.9\times 10^{-4},\;\epsilon_{\eta }=9.7\times 10^{-4}.$$ (59)

## 6 Related Works

The problem of uncertainty propagation through nonlinear dynamical systems subject to stochastic excitation is given by the FPE, which describes the evolution of the PDF, and has been extensively studied in the literature. A number of numerical methods such as the path integral technique (Wehner and Wolfer, 1983; Subramaniam and Vedula, 2017), the finite difference and the finite element method (Kumar and Narayanan, 2006; Pichler et al., 2013) have been proposed to solve the FPE.

These methods inevitably require mesh or associated transformations, which increase the amount of computation. The problem becomes worse when the system dimension increases. To maintain accuracy in traditional discretization based numerical methods, the number of degrees of freedom of the approximation, i.e. the number of unknowns, grows exponentially as the dimensionality of the underlying state-space increases.

On the other hand, the Monte Carlo method, that is common for such kind of problems (Kikuchi et al., 1991; Küchlin and Jenny, 2017), has slow rate of convergence, causing it to become computationally burdensome as the underlying dimensionality increases. Hence, the so-called curse of dimensionality fundamentally limits the use of the FPE for uncertainty quantification in high dimensional systems.

In recent years, low-rank tensor approximations have become especially popular for solving multidimensional problems in various fields of knowledge (Cichocki et al., 2016). However, for the FPE, this approach is not yet widely used. We note the works (Dolgov et al., 2012; Sun and Kumar, 2014; Sun and Kumar, 2015; Dolgov, 2019; Fox et al., 2020) in which the low-rank TT-decomposition was proposed for solution of the multidimensional FPE. In these works, the differential operator and the right-hand side of the system are represented in the form of TT-tensor. Moreover, in paper (Dolgov et al., 2012) the joint discretization of the solution in space-time is considered. The difference of our approach from these works is its more explicit iterative form for time integration, as well as the absence of the need to represent the right hand side of the system in a low-rank format, which allows to use this approach in machine learning applications.

## 7 Conclusion

In this paper we proposed the novel numerical scheme for solution of the multidimensional Fokker–Planck equation, which is based on the Chebyshev interpolation and spectral differentiation techniques as well as low rank tensor approximations, namely, the tensor train decomposition and cross approximation method, which in combination make it possible to drastically reduce the number of degrees of freedom required to maintain accuracy as dimensionality increases.

The proposed approach can be used for the numerical analysis of uncertainty propagation through nonlinear dynamical systems subject to stochastic excitations, and we demonstrated its effectiveness on a number of multidimensional problems, including Ornstein-Uhlenbeck process and dumbbell model.

As part of the further development of this work, we plan to conduct more rigorous estimates of the convergence of the proposed scheme, as well as formulate a set of heuristics for the optimal choice of number of time and spatial grid points and tensor train rank. Another promising direction for further research is the application of established approaches and developed solver to the problem of density estimation for machine learning models.

## Data Availability

Program code, input data and calculation results can be found here: https://github.com/AndreiChertkov/fpcross

## Footnotes

1 Vectors and matrices are denoted hereinafter by lower case bold letters (a,b,c,…) and upper case letters (A,B,C,…) respectively. We denote the $\left(i_{1},i_{2}\right)$ th element of an $N_{1}\times N_{2}$ matrix *A* as $A\left[i_{1},i_{2}\right]$ and assume that $1\leq i_{1}\leq N_{1}$, $1\leq i_{2}\leq N_{2}$. For vectors we use the same notation: a[i] is the *i*-th element of the vector a (i=1,2,…,N). In addition, for a compact representation of an *i*-th (i=1,2,…,d, where d≥1) element of a vector function $f=\;{\left[f_{1},f_{2},\ldots ,f_{d}\right]}^{⊤}$, we will use the notation $f_{i}$, which means $f_{i}\left(\;\cdot \;\right)=f_{i}\left(\;\cdot \;\right)\left[i\right]$.

2 The code is publicly available from https://github.com/AndreiChertkov/fpcross.

3 We use notation $I_{k}$ for the k×k (k=1,2,…) identity matrix.

4 We suppose that for each spatial dimension the variable *x* varies within [−1, 1]. In other cases, an appropriate scaling can be easily applied.

5 By tensors we mean multidimensional arrays with a number of dimensions *d* (d≥1). A two-dimensional tensor (d=2) is a matrix, and when d=1 it is a vector. For tensors with d>2 we use upper case calligraphic letters (A,ℬ,C,…). The $\left(n_{1},n_{2},\ldots ,n_{d}\right)$ th entry of a *d*-dimensional tensor $A\in {\mathbb{R}}^{N_{1}\times N_{2}\times \ldots \times N_{d}}$ is denoted by $A\left[n_{1},n_{2},\ldots ,n_{d}\right]$, where $n_{k}=1,2,\ldots ,N_{k}$ (k=1,2,…,d) and $N_{k}$ is a size of the *k*-th mode, and mode-*k* slice of such tensor is denoted by $A\left[n_{1},\ldots ,n_{k-1},:,n_{k+1},\ldots ,n_{d}\right]$.

6 Note that for the case $N_{1}=N_{2}=\ldots =N_{d}\equiv N_{0}$, we have only one matrix $D_{1}=D_{2}=\ldots =D_{d}\equiv D_{0}\in {\mathbb{R}}^{N_{0}\times N_{0}}$ which greatly simplifies the computation process.

7 By the full format tensor representation or uncompressed tensor we mean the case, when one calculates and saves in the memory all tensor elements. The number of elements of an uncompressed tensor (hence, the memory required to store it) and the amount of operations required to perform basic operations with such tensor grows exponentially in the dimensionality, and this problem is called the curse of dimensionality.

8 All basic operations in the TT-format are implemented in the ttpy python package https://github.com/oseledets/ttpy and its MATLAB version https://github.com/oseledets/TT-Toolbox.

9 An exact TT-representation exists for the given full tensor $\hat{ℛ}$, and TT-ranks of such representation are bounded by ranks of the corresponding unfolding matrices (Oseledets, 2011). Nevertheless, in practical applications it is more useful to construct TT-approximation with a prescribed accuracy $\epsilon_{TT}$, and then carry out all operations (summations, products, etc) in the TT-format, maintaining the same accuracy $\epsilon_{TT}$ of the result.

10 Note that in Algorithm 4 we use the solution of the convection part from the previous time step (k−1) as an initial guess $W_{0}$ for CAM on the next time step (*k*). As it was found empirically It may seem more logical to use the solution of the diffusion part from the same time step (*k*) as an initial guess, but we found empirically that it leads to higher TT-ranks of the result.

11 Actual links to the corresponding Colab notebooks are available in our public repository https://github.com/AndreiChertkov/fpcross.

12 Hereinafter, we present effective TT-rank of the computation result. For TT-tensor $X\in {\mathbb{R}}^{N_{1}\times N_{2}\times \ldots \times N_{d}}$ with TT-ranks $R_{0},R_{1},\ldots ,R_{d}$ ($R_{0}=R_{d}=1$) the effective TT-rank $\hat{R}$ is a solution of quadratic equation $N_{1}\hat{R}+\sum_{\alpha =2}^{d-1}N_{\alpha }{\hat{R}}^{2}+N_{d}\hat{R}=\sum_{\alpha =1}^{d}N_{\alpha }R_{\alpha -1}R_{\alpha }.$ The representation with a constant TT-rank $\hat{R}$ (${\hat{R}}_{0}=1$, ${\hat{R}}_{1}={\hat{R}}_{2}\ldots ={\hat{R}}_{d-1}=\hat{R}$, ${\hat{R}}_{d}=1$) yields the same total number of parameters as in the original decomposition of the tensor X.

13 This choice of parameters corresponds to the problem of polymer modeling from the work (Venkiteswaran and Junk, 2005). In the corresponding model, the molecules of the polymer are represented by beads and interactions are indicated by connecting springs. Accordingly, for the case of only two particles we come to the dumbbell problem, which can be mathematically written in the form of the FPE.

14 As values for comparison, we used the result of the most accurate calculation from work (Dolgov et al., 2012), within which $\hat{\psi }\left(t=10\right)=2.071143$, and $\hat{\eta }\left(t=10\right)=1.0328125$.
